# Equine strongyle communities are constrained by horse sex and species dipersal-fecundity trade-off

**DOI:** 10.1186/s13071-018-2858-9

**Published:** 2018-05-02

**Authors:** Guillaume Sallé, Sławomir Kornaś, Marta Basiaga

**Affiliations:** 1 UMR1282 INRA/Université de Tours Infectiologie et Santé Publique, F-37380 Nouzilly, France; 2 Department of Environmental Zoology, Institute of Animal Sciences, University of Agriculture in Krakow, al. Mickiewicza 24/28, 30-059, Krakow, Poland

**Keywords:** Horse, Cyathostomes, Strongyles, Ecology, Sex, Nematode, Diversity

## Abstract

**Background:**

Equine strongyles are a major health issue. Large strongyles can cause death of horses while cyathostomins (small strongyles) have shown increased resistance to anthelmintics worldwide. Description of strongyle communities have accumulated but little is known about the diversity of these communities and underpinning environmental factors.

**Methods:**

Strongyles were recovered after ivermectin treatment from 48 horses located in six premises in Poland. Correlation between previously published species fecundity and the observed relative abundance and prevalence were estimated. Significance of horse sex was determined at the species level (prevalence, relative abundance) and at the community level (species richness and dissimilarity between communities).

**Results:**

Strongyle species fell into two groups, contrasted by their prevalence and relative abundance. Six to nine horses were necessary to sample at least 90% of strongyle community diversity, providing a minimal cut-off to implement sampling trial in the field. Strongyle communities entertained a network of mostly positive interactions and species co-occurrence was found more often than expected by chance. In addition, species fecundity and prevalence were negatively correlated (Pearson’s *r* = -0.71), suggesting functional trade-offs between species dispersal abilities and fecundity. This functional trade-off may underpin species coexistence. Horse sex was also a significant constraint shaping strongyle communities. Indeed, mares generally displayed more similar strongyle communities than stallions (*P* = 0.003) and *Cylicostephanus calicatus* was more abundant in stallions suggesting sex-specific interactions (*P* = 0.006).

**Conclusions:**

While niche partitioning is likely to explain some of the positive interactions between equine strongyle species, coexistence may also result from a functional trade-off between dispersal ability and fecundity. There is significant evidence that horse sex drives strongylid community structure, which may require differential control strategies between mares and stallions.

**Electronic supplementary material:**

The online version of this article (10.1186/s13071-018-2858-9) contains supplementary material, which is available to authorized users.

## Background

Grazing horses are infected by a wide variety of intestinal strongyle parasites belonging to the subfamilies Strongylinae and Cyathostominae [1]. Among the Strongylinae, *Strongylus vulgaris* has been identified as a major cause of colic in horses [2]. This results from the accumulation of larval stages in the cranial mesenteric artery that leads to an arteritis responsible for fatal intestinal infarction [2]. However, modern anthelmintic treatments have dramatically decreased its prevalence [3]. Cyathostominae (cyathostomes or small strongyles) form a wider tribe than equine Strongylinae, encompassing 51 species, 40 of which infect horses [1]. Unlike the Strongylinae, these nematodes can reach prevalences up to 100% in horses [4]. Cyathostomes have been associated with milder clinical signs like poor hair coat and weight loss [5], although the massive emergence of larval stages from the large intestine mucosa where they reside can lead to larval cyathostominosis cases, characterised by diarrhoea and protein-losing enteropathy [6]. Failure to control larval cyathostominosis can also lead to the death of affected horses in at least a third of cases, as suggested by a report on 15 clinical cases in the UK [7]. More importantly, cyathostomes have been involved in multiple reports of anthelmintic failures throughout the world thus representing a major issue in equine medicine [8–11].

Despite the wide variety of species, cyathostome communities are consistently described as a core assemblage of 10–13 species [4, 12–16]. While this has been frequently reported, only scant knowledge has been gathered about the drivers underpinning strongyle biodiversity or community assemblage. Nonetheless, some efforts have been made to characterise the consequences of deworming treatments on strongyle diversity as an attempt to better understand widespread anthelmintic failures. First, a reduced diversity in strongyle communities was observed in Ukrainian farms applying frequent deworming [17]. Secondly, investigations of strongyle community structure in horse populations after anthelmintic treatment found that the first species to reappear represented a limited subset of total community [18, 19]. *Cylicocyclus nassatus* and *Cylicostephanus longibursatus* were consistently found among the major species after a combination of pyrantel followed by fenbendazole treatment [18] or after ivermectin treatment [19]. Another study also suggested that these two species were driving the drug resistance phenotype [20, 21]. However, these observations might be confounded by the reported variation in the number of eggs found *in utero* across strongyle species [22], dispersal ability (measured by the prevalence of infection) or relative abundance [12, 13, 16]. Indeed, these latter properties may affect the post-treatment community structure, e.g. the most abundant species contributing most to the post-treatment community, without any genuine association with anthelmintic susceptibility.

Some insights into the structure of equine strongyle communities have been provided by a report focusing on pair-wise correlations between strongyle species abundance [23]. This analysis revealed negative interactions between major species, suggesting competition might be involved [23]. In addition, an attempt to bring together environmental factors with equine strongyle diversity in Ukraine concluded that foals and horses older than 16 years displayed higher species richness [24]. However, species richness is only a limited facet of the actual biodiversity as it does not account for the relative proportions of every species found. Furthermore, the impact of host sex was not considered [24].

Host sex effect is a major driver of helminth community assemblages, male mammals being generally more frequently and more heavily parasitized [25, 26], although some discrepancies exist to this general pattern [27]. In horses, no clear consensus has been reached so far. The analysis of necropsy reports in Australia found differential prevalence and abundance of 13 endoparasites between the two sexes [28]. However, no general trend emerged as females were more heavily infected by *Cyathostomum pateratum* but less frequently infected by *Cylicodontophorus bicoronatus*, *Cylicocyclus insigne* and *Cylicocyclus elongatus* than stallions and geldings [28]. Study of faecal egg count in a feral horse population reported a spatially contrasted polarity of the sex effect, stallions being more affected than females on one side of the island while the opposite trend was found on the other side [29]. Other studies reported higher faecal egg count in geldings or could not evidence any difference [30, 31].

In this study, we analysed strongyle communities recovered from 48 horses in Poland to investigate species community structure and diversity, the link between previously published fecundity estimates and species dispersal ability and to assess how they were affected by horse sex.

## Methods

### Study population

Strongyles were collected in spring 2011 and 2012 from 48 horses scattered across six premises from Poland (Additional file 1: Table S1). Premises had been enrolled for an ivermectin efficacy test. Horses were selected based on their faecal egg count before treatment to allow for strongyle recovery. The study population was composed of 37 mares and 11 stallions. The Małopolska breed accounted for most of the sampled horses (*n* = 25), in addition to some Hucul (*n* = 6) and pure blood Arabian horses (*n* = 17) that were found in only two sites (Additional file 1: Table S1). Two thirds of the individuals were born before 2007 (Additional file 1: Table S1).

Within each premise, the number of sampled horses ranged from 3 to 18 individuals (Table 1, Additional file 1: Table S1).

**Table 1:** Premise features and ecological properties of recovered strongyle communities. Average species richness (number of recovered strongyle species) and Shannon’s index of diversity are provided for each premise

Premise	Type	No. of horses (*n*)	Mares	Stallions	Species richness ± SD	Shannon index ± SD
1	Stud farm	18	18	0	12.17 ± 2.04	2.07 ± 0.17
2	Riding school	14	8	4	9.86 ± 3.74	1.59 ± 0.48
3	Riding school	3	2	1	6.00 ± 0.01	1.46 ± 0.23
4	Riding school	3	1	2	6.67 ± 1.53	1.43 ± 0.12
5	Riding school	3	0	3	11.67 ± 6.51	1.75 ± 0.38
6	Riding school	7	6	1	11.00 ± 2.16	1.90 ± 0.14
Total		48	35	11	10.6 ± 3.4	1.8 ± 0.38

*Abbreviation*: *SD* standard deviation

Parasite control strategy in every location relied on ivermectin treatments administered in March (or April) and October, before and after pasture season, respectively.

### Strongyle collection and identification procedure

The species composition of strongylid nematodes was performed by collecting 500 g of faecal material expelled 24 h after ivermectin treatment following the strategy of other trials [14, 32]. Subsequently, samples were rinsed with tap water on 250 μm mesh sieves and examined under stereomicroscope for the presence of strongylids. Collected nematodes were fixed in 70% ethyl alcohol with 5% glycerine additive to clear their body structure. Strongylids were identified according to previously published morphological keys [1].

Faecal egg count before treatment was performed following a modified McMaster technique [33] on 5 g of faecal material, diluted in 70 ml of a flotation solution (NaCl, d = 1.18), with a sensitivity of 50 eggs/g.

### Community structure analyses

Statistical analyses were implemented with R v 3.4.1 [34]. Unless stated otherwise, any *P*-value below 5% was regarded as significant. Strongyles recovered from each horse are hereafter referred to as a community. Within each community, species intensity refers to the number of worms recovered from a given horse, while species relative abundance in a given community corresponds to the proportion of worms found relative to the total worm burden. Species prevalence was defined at the premise level as the proportion of horses infected by a given strongyle species.

Species fecundity may contribute to observed relative abundance and prevalence. To investigate this point, the correlation between previously reported number of eggs *in utero* recovered from strongyle females [22] and observed species abundance and prevalence were estimated (*rcorr* function of the *Hmisc* package v.4.0-3 [35]). Species fecundity estimates were log-transformed to obtain a linear relationship between variables.

Our sampling procedure involved a limited amount of faecal material which may have biased our prevalence and abundance estimates. Therefore, our observations were compared against a dataset [36] of previously published equine strongyle species prevalence (*n* = 29 papers) and relative abundances (*n* = 14 papers) provided as Additional file 2: Table S2. These studies spanned all continents and involved various methodologies (necropsy, deworming), thus limiting confounding factors linked to climatic conditions or sampling methods. For each paper, species relative abundance and prevalence were computed from the data if not already provided. These individual estimates were subsequently averaged across studies for comparison with the observed values from our 48 horses.

Community diversity analyses were run using the *vegan* package v. 2.4-3 [37]. Species richness was defined as the total number of species recovered from each horse. Species diversity within each horse, corresponding to Whittaker’s α-diversity [38], was measured by the Shannon’s index [39] defined as:

$$ H=\sum \limits_i^n{p}_i\log \left({p}_i\right) $$

where *p*_*i*_ is the proportional abundance of species *i*. This was implemented with the *diversity* function of the *vegan* package [37]. Normality of species richness was tested by a Shapiro-Wilk test.

Species turnover can occur along environmental gradients, hence resulting in variation among individual communities from a given group. This measure of dissimilarities between communities was originally coined as β-diversity [38], and will contribute only marginally to the global diversity if individual communities show little or no variation among groups. Difference in β-diversity among environmental factors (location, horse age and horse sex) was tested following a multivariate framework [40, 41], implemented in the *adonis* function of the *vegan* package [37]. This framework measures β-diversity as the distance of every community to the average group community, determined as the centroid of a principal components analysis of community structures [40].

Non-random species co-occurrence was tested following a probabilistic framework that compares observed species frequencies to the expected frequencies under species independence (discarding species pairs when the expected number of co-occurrence is less than 1) [42, 43]. Species interactions could also result in positive or negative feedbacks that could impact on species relative abundances. To test for these interactions, species counts were regressed upon other species intensity in a pairwise manner after accounting for environmental variables (horse sex and sampling site) and by means of a Poisson regression. For this analysis, the 13 species with more than 200 individuals across horses were considered, as failure to do so results in strong positive associations for the minor species present. Because of the high number of statistical tests (*n* = 105), a Bonferroni correction was applied and a *P-*value below 0.05/105 = 5 × 10^-4^ was considered significant. Significant pairwise regression estimates (*P* < 5 × 10^-4^) were used to generate a species network, visualized with the *ggnet* package [44]. The network uses a force-directed algorithm from the matrix of interactions. Nodes act as repelling objects that are organized to minimize forces in the network.

### Host sex effect on species relative abundance and prevalence

The impact of host sex on strongyle abundance (worm counts) and prevalence was investigated for the 11 core strongyle species, i.e. most abundant and most prevalent. *Cyathostomum catinatum* showed the highest prevalence (47 horses infected) and was not included in the prevalence data analysis as not enough variability was present to assess factors of variation. To account for the data structure (Additional file 1: Table S1), these effects were investigated using only the data from premises where horses of each gender were present (premises 2, 3, 4 and 6; *n* = 27 horses). Worm counts and species prevalence were modelled using a negative binomial and logistic regression, respectively.

Samples were recovered in 2011 and 2012, hence introducing a putative confounding annual variation. This was tested in two ways. First, Pearson’s correlation between prevalence and relative abundance measured in 2011 and 2012 were estimated to support their congruence. Secondly, worm counts and species prevalence were regressed on a universal year effect shared across species and a species-specific inter-annual variation.

After ruling out a significant inter-annual variation, worm counts and species prevalence were modelled fitting the premise, strongyle species, horse sex and their interaction as fixed effects, and horse age as a covariable:

$$ {\displaystyle \begin{array}{l}y\sim Strongyle\ species+ Horse\  age+ Horse\  sex+ Strongyle\ species\times \\ {} Horse\  sex+\mathit{\Pr} emise\end{array}} $$

where y stands for species count or prevalence accordingly.

A linear regression analysis was applied to Shannon’s index values to evaluate variation between premises and horse ages and sexes. For every model, the sex effect on species relative abundance and prevalence was estimated across premises (19 mares and 8 stallions). The premise effect accounted for both management and breed type differences.

## Results

### Strongyle dispersal abilities were contrasted and inversely correlated with their known fecundity estimates

Considered parasite populations were all ivermectin-susceptible as post-treatment FEC were back to 0 in every horse. On average, 608.7 worms per horse were collected, ranging from 111 to 2884 (Additional file 1: Table S1). Overall, 23 species were found across premises (Additional file 1: Table S1), with average species richness ranging from 6 to 12 species (Table 1).

Between 4 and 20 species could be recovered at the horse level (Additional file 1: Table S1). The male to female worm ratio was 0.58 on average (Additional file 3: Table S3). Total worm count was not significantly correlated with pre-treatment faecal egg count (Spearman’s *r*_(48)_ = 0.22, *P* = 0.13).

**Fig. 1:**
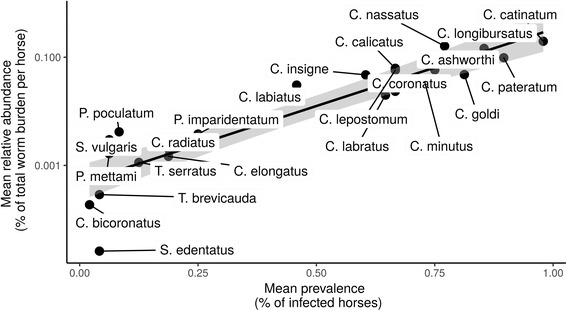
Relationship between strongyle species prevalence and relative abundance. Mean relative abundance is expressed as the fraction of a given species relative to the total number of strongyles recovered across the 48 horses involved in this study. Mean prevalence represents the percentage of horses infected by a given species. Each dot stands for one species. The black line represents the regression line between the two variables, with associated 95% confidence interval shown as a grey area

*Cyathostomum catinatum* and *C. bicoronatus* were the most and the least abundant species, respectively; worms of each species summing up to 5462 and one worms recovered across communities, respectively. *Strongylus vulgaris* was encountered in only 3 horses. Relative species abundance across communities could distinguish between two groups of species, being either highly abundant and prevalent (Fig. 1) or with lower dispersal ability and minor contribution to communities (Fig. 1). *Cyathostomum catinatum*, *C. nassatus* and *C. longibursatus* were the three most abundant species (accounting for 21.1, 17.8 and 13.7% of the total worm burden, respectively) and were found in three quarters of horses (*n* = 47, 37 and 41 horses, respectively; Fig. 1). These observations showed strong congruency with previously published estimates (Pearson’s *r*_(19)_ = 0.83, *P* < 0.0001 and *r*_(21)_*=* 0.90, *P* < 0.0001 for prevalence and relative abundance estimates, respectively). This independent dataset also supported the outlying contribution of *C. catinatum*, *C. nassatus* and *C. longibursatus*, that infected 67.3, 68.9 and 72.1% of horses and accounted for 17.4, 18.2 and 16.8% of total worm burden across studies.

**Fig. 2:**
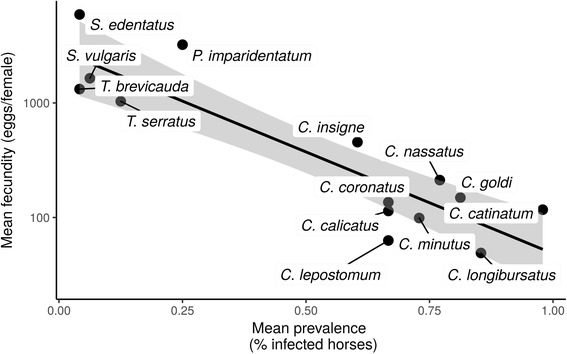
Relationship between estimated strongyle prevalence and known fecundity estimates. Previously published estimates of strongyle species fecundity (measured as the number of eggs found in female strongyle *in utero*) were plotted against species prevalence across monitored horses (measured as the fraction of horses infected by a given species). Each dot stands for a given strongyle species. The regression line between the two variables is shown as well the 95% confidence interval (grey area)

Pearson’s correlations between published estimates of worm fecundity and measured species abundance and prevalence were estimated to determine whether fecundity could underpin better dispersal abilities. These correlations suggested that fecundity was negatively correlated with species prevalence (Pearson’s *r*_(14)_ = -0.71, *P* = 0.004; Fig. 2) and that the same trend held for species relative abundance (Pearson’s *r*_(14)_ = -0.48, *P* = 0.08; Additional file 4: Figure S1). A significant correlation was found between fecundity and relative abundance when considering only female worm counts (Pearson’s *r*_(14)_ = -0.57, *P* = 0.03).

A similar pattern was found when using the 29 previously published prevalence estimates (Pearson’s *r*_(13)_ = -0.86, *P* < 0.0001), but the relationship with 14 published species relative abundance was not significant (Pearson’s *r*_(10)_ = -0.45, *P* = 0.19).

**Fig. 3:**
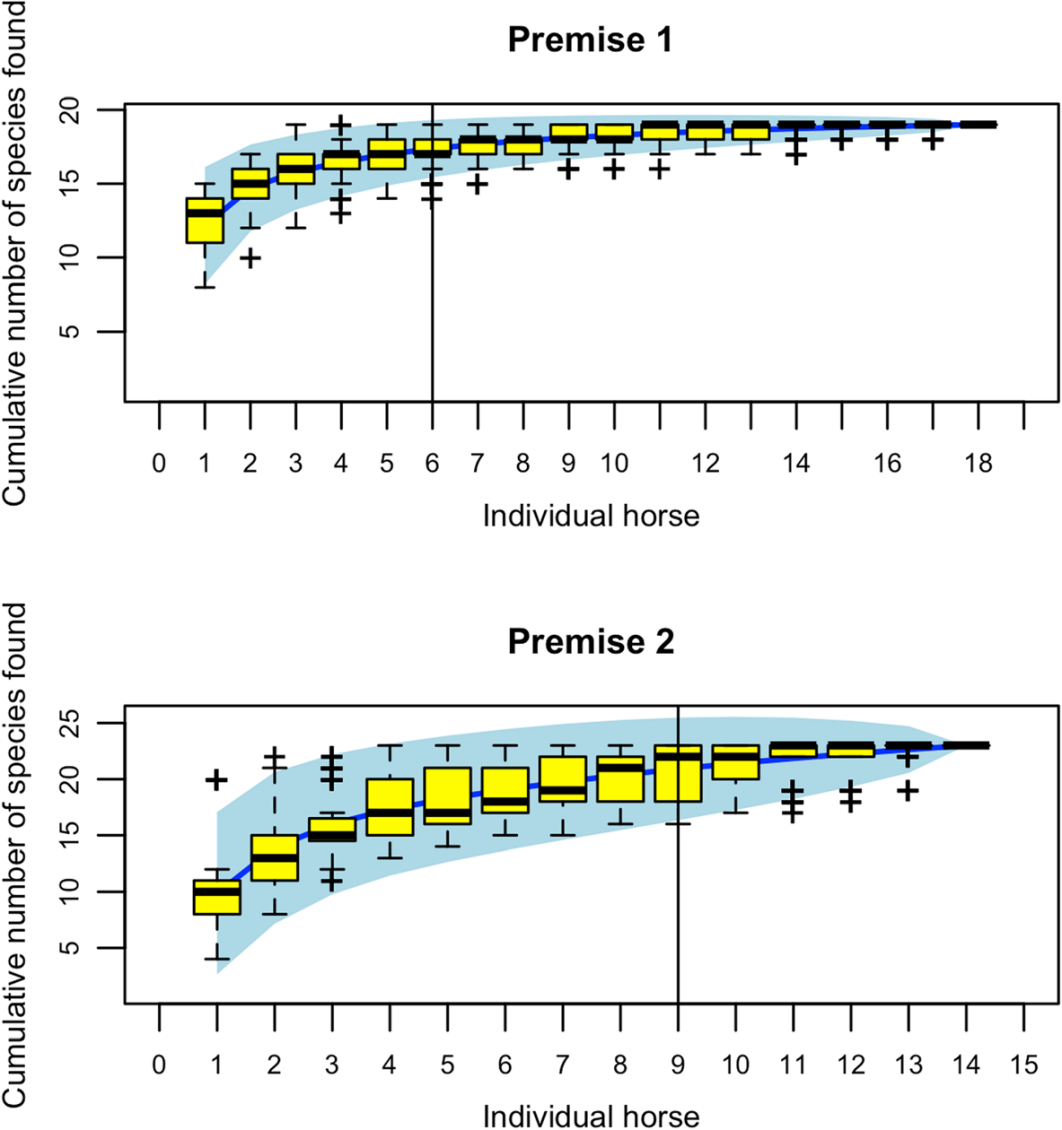
Species accumulation curves for the two most populated premises. For these two premises, the sampling effort (number of horses to sample) required to detect a given number of species (y-axis) is plotted as a blue line with 95% confidence interval (blue area). Box-plots stand for random variation measured by permutations. Vertical lines assess the number of horses needed to detect 90% of total species in a given premise

Species richness accumulation curves suggested that the minimal sampling effort had to be comprised between six and nine horses to detect 90% of total species present in one location (Fig. 3).

### Communities were structured by a network of mostly positive interactions

**Fig. 4:**
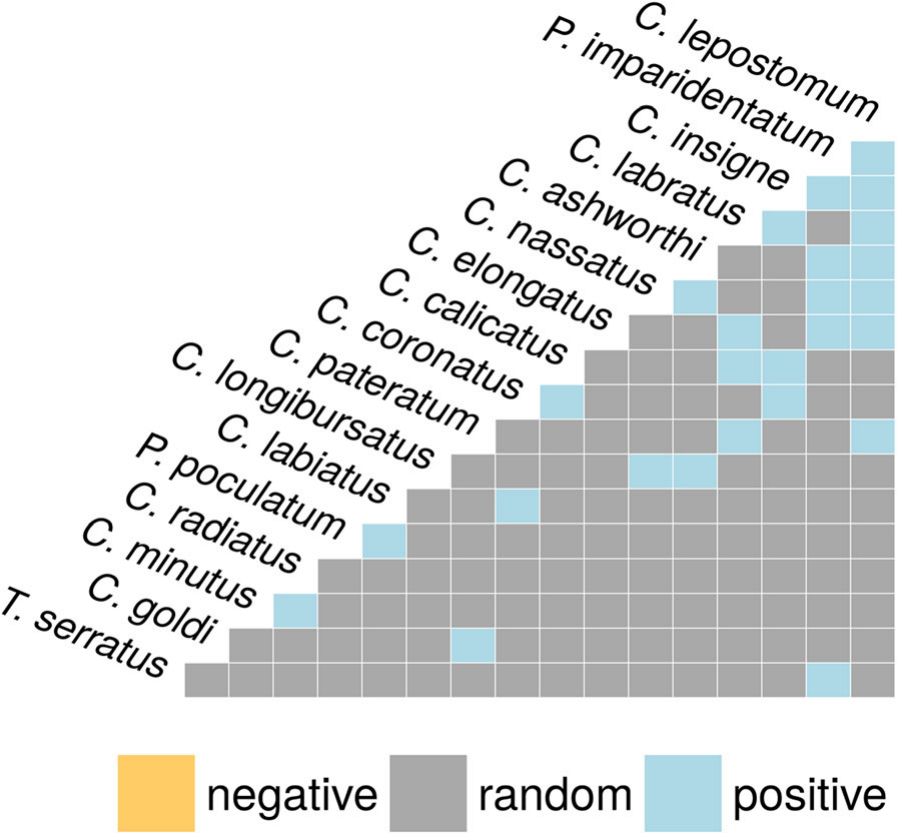
Strongyle species co-occurrence. A pairwise matrix of co-occurrence between species co-occurring more than once. Grey boxes indicate that species pairs occurred randomly, whereas 26 pairs co-occurred significantly more often than expected (blue boxes).

Species co-occurrence analysis was dominated by random co-occurrences that represented 87% of the 200 tested combinations (Fig. 4, Additional file 5: Table S4). Nonetheless, a few species pairs seemed to positively interact with each other (*n* = 26, Fig 4, Additional file 5: Table S4), including some core species, e.g. *C. longibursatus*-*C. nassatus* (*P* = 0.04) or *Cyathostomum pateratum*- *Cylicostephanus goldi* (*P* = 0.04).

**Fig. 5:**
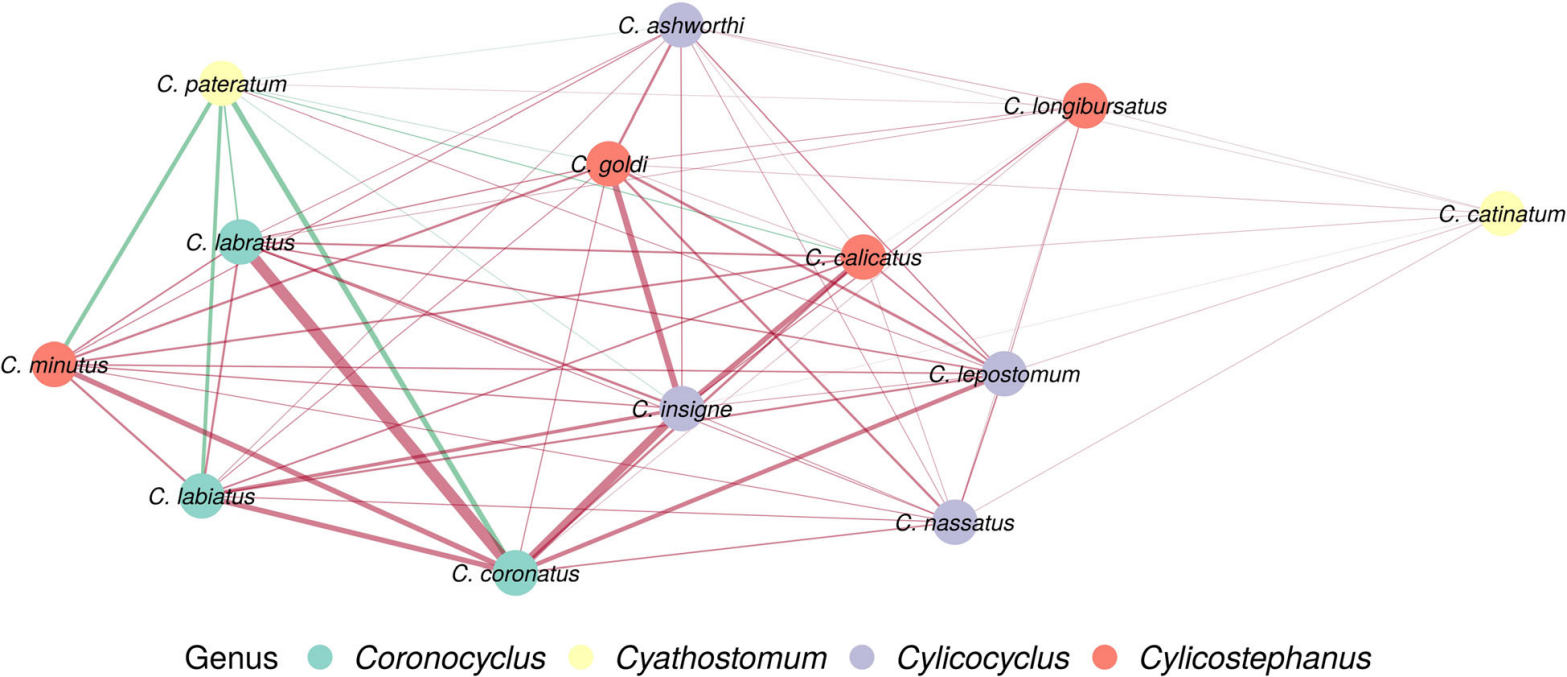
Interactions network based on relative abundances for recovered strongylid species. A network, drawn using a force-directed algorithm from the matrix of interactions estimated by pairwise Poisson regression on species counts after accounting for environmental effects. To avoid spurious positive associations due to low counts, the analysis was restricted to the only species totalling 200 worms overall. Nodes act as repelling objects that are organized to minimize forces in the network. Each node represents a species and is coloured according to the genus it belongs to. Connected nodes entertain significant association after Bonferroni correction for multiple testing (*P* < 5 × 10^-4^). Edges are coloured to reflect the correlation sign (red if positive, green if negative) and their widths mirror the association intensity (the thicker, the bigger absolute effect)

Significant pairwise interactions (Additional file 6: Table S5) were used to build a network between *Coronocyclus*, *Cyathostomum*, *Cylicostephanus* and *Cylicocyclus* species (Fig. 5) that was also mostly driven by positive interactions between species intensities (Fig. 5). Notably, *C. pateratum* was the only species whose intensity varied negatively with other species, except for *C. longibursatus* and *Cylicocyclus lepostomum* (Fig. 5). Three of the best dispersers (*C. catinatum*, *C. nassatus* and *C. longibursatus*) seated on the edge of the network, with weak links to other species, suggesting that variation in their intensity was not impacted by other species. On the contrary, *Coronocyclus* species, i.e. *C. coronatus*, *C. labratus* and *C. labiatus*, were more tightly connected, supporting similar behaviors.

### Horse gender affects dissimilarity between strongyles communities and species abundance

Little inter-annual variation was found as the correlation for prevalence and relative abundances across the two years were 0.89 and 0.91, respectively (Pearson’s *r*_(23)_, *P* < 0.0001 in both cases). In addition, neither a universal inter-annual effect shared across species (Wald test *χ*^2^ = 1.35, *df* = 1, *P* = 0.25 for prevalence; Wald test *χ*^2^ = 0.28, *df* = 1, *P* = 0.59 for relative abundance) nor a species-specific annual variation (Wald test *χ*^2^ = 14.48, *df* = 10, *P* = 0.15 for prevalence; Wald test *χ*^2^ = 11.01, *df* = 10, *P* = 0.36 for relative abundance) were found.

Strongyle community diversity ranged from 0.6 to 2.6 at the horse level but was more consistent at the premise level with average values between 1.43–2.07 (Table 1). No significant variation in species α-diversity was found between premises (*F*_(3, 21)_ = 2, *P* = 0.15).

Neither horse age (*F*_(1, 21)_ = 2.33, *P* = 0.14) nor horse sex affected significantly community diversity (*F*_(1, 21)_ = 0.33, *P* = 0.57), suggesting that α-diversity was equivalent between mares and stallions.

An analysis of community dissimilarity was performed to assess the extent of species turnover across horse gender, horse age and premises. Strongyle communities structure was similar across horse ages (*F*_(1, 21)_ = 1.60, *P* = 0.24) but significantly varied between premises (*F*_(3, 21)_ = 2.38, *P* = 0.05) and were also significantly more homogeneous in mares than in stallions (*F*_(1, 21)_ = 8.72, *P* = 0.006).

**Fig. 6:**
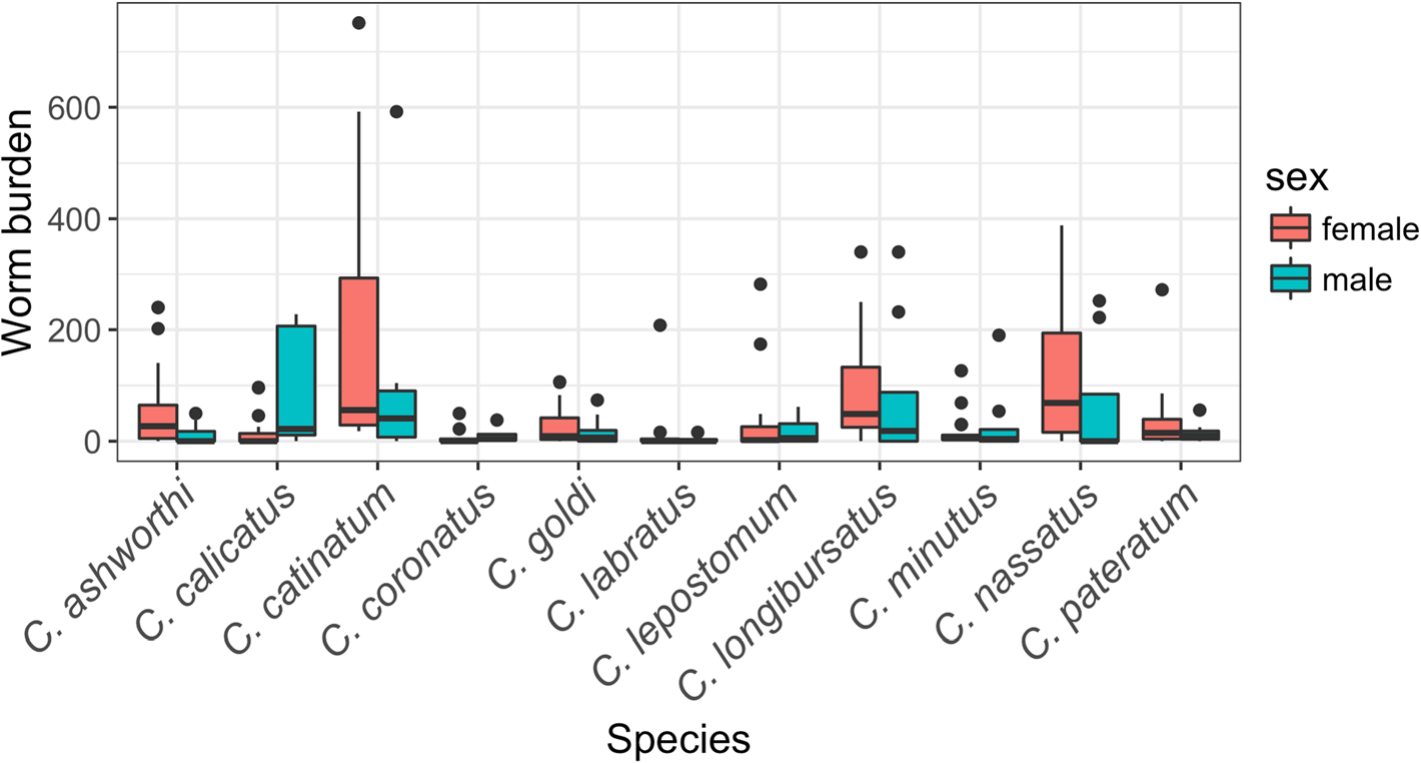
Strongyle abundance variation according to horse gender. Boxplots of strongyle counts are plotted against horse gender (red for females and blue for males) across premises. Within each box, the midline shows the median, and the lower and upper box edges delimit the 25 and 75% quantiles, respectively. Dots represent outliers (1.5-fold higher than the 75% quantile). Of note, is the higher abundance for *C. calicatus* in males

No systematic horse sex driven differences in strongyle species abundance was identified across strongyle species by the binomial regression approach (Wald test *χ*^2^ = 0.8, *df* = 1, *P* = 0.37, Fig. 5). However, a significant interaction was found between horse gender and strongyle species abundance (Wald test *χ*^2^ = 23.4, *df* = 10, *P* = 0.009). Notably, *C. calicatus* was significantly more abundant in stallions than in mares (*t*_(271)_ *=* 2.77, *P* = 0.006), whereas the opposite trend (non-significant) was generally found for other species (Fig. 6, Additional file 7: Table S6). This pattern was also conserved when comparing *C. calicatus* abundance between premise 1 (14.8 worms/horse on average; mares only) and premise 5 (107 worms/horse on average; stallions only) even though this difference could not be disentangled from a premise-associated effect.

Strongyle dispersal ability measured by the prevalence of each species did not vary significantly between mares and stallions, although *C. nassatus* was found slightly more often in mares (*t*_(271)_ *=* -1.94, *P* = 0.05, Fig. 6, Additional file 7: Table S6).

## Discussion

This study focused on the structure of equine strongyle communities under eastern Europe climatic conditions. The data suggested that reported species fecundity, approximated by the number of eggs *in utero*, was negatively correlated with species prevalence. This relationship was found both in our dataset and across previously published prevalence reports. In addition, horse sex had a statistically significant effect on strongyle community structure and diversity.

Numerous reports on equine strongyle community structure have already evidenced the bimodal pattern of their assemblages, with five to ten highly prevalent and abundant species and additional minor species [4, 12–16]. Our data also highlighted the same split between core and satellite species [45], with a set of three successful dispersers, i.e. *C. catinatum*, *C. nassatus* and *C. longibursatus*, which were found in 75% of horses and represented between 13–21% of the total worm burden. This pattern was also corroborated by estimates from available literature.

Not only corroborating the known literature, this finding also supported the chosen sampling strategy [14, 32]. First, only a limited amount of faecal material was taken from each horse. This could have biased downwards worm counts, eventually resulting in increasing the missing rate of some species. However, it seems that our prevalence and relative abundance fit the pattern observed across previous reports that relied on necropsy or deworming for worm collection. Secondly, a single sampling 24 h after treatment might have biased the sampling efficiency downwards as it has been suggested that a significant proportion of parasite material could still be recovered after 36 and up to 60 h [14]. From our results, some species reported as minor in the present study (*S. edentatus*, *S. vulgaris*, *T. serratus* and *C. elongatus*) may have suffered from the sampling strategy as most of them were expelled 48 h after treatment onwards in a previous trial [14]. Notably, this prevents any firm conclusion regarding the minor contribution of *S. vulgaris* to the strongyle communities, even though displayed prevalence levels were in agreement with other reports from regularly dewormed horses [46, 47]. Nevertheless, community structure and species richness estimates were similar to previous reports under eastern European conditions [14, 17], supporting the implemented sampling method.

Under the considered conditions, a limited number of horses were required to sample 90% of the strongyle community diversity. This is of particular relevance for the implementation of field sampling where necropsy is not an option and minimal interference with premise activity are expected.

Although the overall structure of strongyle communities has been well characterized in equids [4, 12–15], little is known about the mechanisms and variation factors underpinning their stability. Remarkably, sampled communities were entertaining positive interactions, both in terms of presence/absence patterns or in their intensity covariation. This finding was in contradiction with previously reported results from an abattoir survey, which highlighted negative interactions between major species [23]. However, the negative interactions mostly occurred between species that were less represented in our present report, i.e. *S. vulgaris* and *S. edentatus* or *Triodontophorus* species [23]. In addition, our analyses were restricted to the best dispersers (species with highest prevalence); hence preventing spurious positive associations that can occur when correlating low counts [48]. Our results were in line with known positive associations between core species [49]. Reports of helminths species co-occurring more often than expected by chance have also been made for human and rodent systems [50]. These positive interactions could arise from niche partitioning between species within horses. The latter could explain for instance, positive feedbacks between *C. goldi* and *C. nassatus*, which mostly occupy the dorsal and ventral colon, respectively [16, 23], but does not support positive interactions between *C. minutus* and *C. calicatus*, which tend to share the same organs [16, 23]. Observed positive interactions may hence suggest that some of the equine strongyle species share mutualistic interactions as already reported for rodent models [50]. However, the negative interactions of *C. pateratum* with other species remains unexplained. It could be the result of competition between species for space or nutrients (exploitation competition); it may also suggest interference between species due to local inflammation mediated by one species and affecting others or cross-reactivity of the host immune system towards multiple species [51].

The existence of functional trade-offs can result in positive associations between species, as demonstrated in plants [52] or fig wasps [53]. Because of the sparsity of hosts and environmental constraints put on eggs and larvae on pasture, strongyle species with best competing abilities, like higher fecundity, may not be able to colonize every available horse. As a result, horses can be colonized by species with lower competing abilities, e.g. lower fecundity. This ultimately leads to species co-existence despite highly differential competing abilities and an apparent trade-off between species fecundity and prevalence. However, our results may suffer from the proxy used for species fecundity. These estimates are based on the number of eggs found *in utero* in female worms, and were generated for a worm population from the USA [22]. A precise assessment of each species egg production rate by means of barcode sequencing may help to better resolve this point.

The negative correlation between species female relative abundance and their inferred fecundity may be related to some crowding effect. It has been described in sheep trichostrongylids [54] and a negative feedback between mature worms and incoming larvae has been suggested in horses [55, 56].

Our results suggested that horse sex was not exerting a universal influence on worm relative abundance across species, but that mares had more consistent strongyle communities. In addition, higher *C. calicatus* worm counts were found in stallions. This finding was already reported in a previous Australian survey, which also evidenced additional host sex interactions for *C. coronatus* and *C. catinatum* [28]. Variation in the qualitative composition of strongyle structure might hence contribute to the lack of clear differences between stallion and mare excretion patterns. However, mechanistic explanations to support a higher species turnover among stallions remain unclear and deserve further exploration.

## Conclusions

This survey provided insights into applied and more fundamental knowledge about equine strongyle communities and how host sex drives their structure and diversity. The bimodal structure of equine strongyle communities split into core and satellite species was confirmed, underscoring the overwhelming contribution of *C. nassatus*, *C. catinatum* and *C. longibursatus*. We also found that less than ten horses were needed to sample most of the diversity, which is particularly relevant for logistically constrained surveys. More fundamentally, it seems that equine strongyle communities are largely structured around a network of positive, if at all, interactions, with the only exception of *C. pateratum*. While mutualistic interactions and niche partitioning could explain this pattern, the observed trade-offs between fecundity and dispersal abilities may also explain this coexistence. Horse sex did not appear to influence the overall worm counts, but a higher species turnover was found among stallions leading to more dissimilar strongyle communities for this gender. While our data provided new insights into the structuring equine strongyle communities, only a limited set of the potential factors shaping these communities have been considered. Further research in this area should make use of recent advances in sequencing technology to increase the sensitivity of worm sampling and also integrate putative interactions with the host gut microbiota.

## Additional files

Additional file 1: **Table S1.** Individual worm counts and associated metadata relative to each of the 48 horses considered: sex, breed, year of birth, premise, premise type, pre-treatment faecal egg count and worm counts for each nematode species. (XLS 72 kb)

Additional file 2: **Table S2.** List of previously published papers on prevalence and relative abundance for equine strongylids. The list of papers (with digital object identifier and link to PubMed reference) used for comparing observed strongyle species prevalence and relative abundance with previous results obtained across different countries is provided. (XLS 60 kb)

Additional file 3: **Table S3.** Species worm counts distinguishing adult female and adult male worms for every horse. Female and male worm counts are provided for each horse (in rows) and strongyle species (in column). (XLS 79 kb)

Additional file 4: **Figure S1.** Relationship between observed strongyle abundance and published fecundity estimates. Previously published estimates of strongyle species fecundity (measured as the number of eggs found in female strongyles *in utero*) are plotted against species relative abundance (measured as the fraction of the total strongyle community within each horse) across monitored horses. Each dot stands for a given strongyle species. The regression line between the two variables is shown as well the 95% confidence interval (grey area). (TIF 737 kb)

Additional file 5: **Table S4.** Co-occurrence probabilities between species. For each strongyle species pair, the observed and expected co-occurences are reported as well as the probability of co-occurrence. In each case, the probability of negative interaction or positive interaction are provided and significant interactions are highlighted in red. (XLS 49 kb)

Additional file 6: **Table S5.** Pairwise regression coefficients between the 13 most abundant species. To test for species interactions in terms of abundance, species counts were regressed upon other species intensity in a pair-wise manner after accounting for environmental variables (horse sex and sampling site) and by means of a Poisson regression. The pairwise regression estimates for the 13 species with than 200 individuals across horses are provided. (XLS 71 kb)

Additional file 7: **Table S6.** Species intensity and prevalence modelling output. Model estimates of the regression of horse age, premise, strongyle species, horse sex and interaction between horse sex and species upon species abundance and prevalence are given. Regression coefficient estimates are provided with associated standard errors and *P*-values. Within each variable, regression coefficients are expressed as the departure from a reference level (*C. catinatum*, mare, premise 2, for the species, horse sex and premise variable, respectively). Interaction estimates correspond to the deviation of a given species in stallions in comparison to mares. Age was fitted as a covariate. (XLS 68 kb)
