# Supplementary figures and images for: Equine strongyle communities are constrained by horse sex and species dipersal-fecundity trade-off
Source: Parasit Vectors. 2018 May 2;11:279. doi: 10.1186/s13071-018-2858-9 (PMC5930759; doi:10.1186/s13071-018-2858-9)

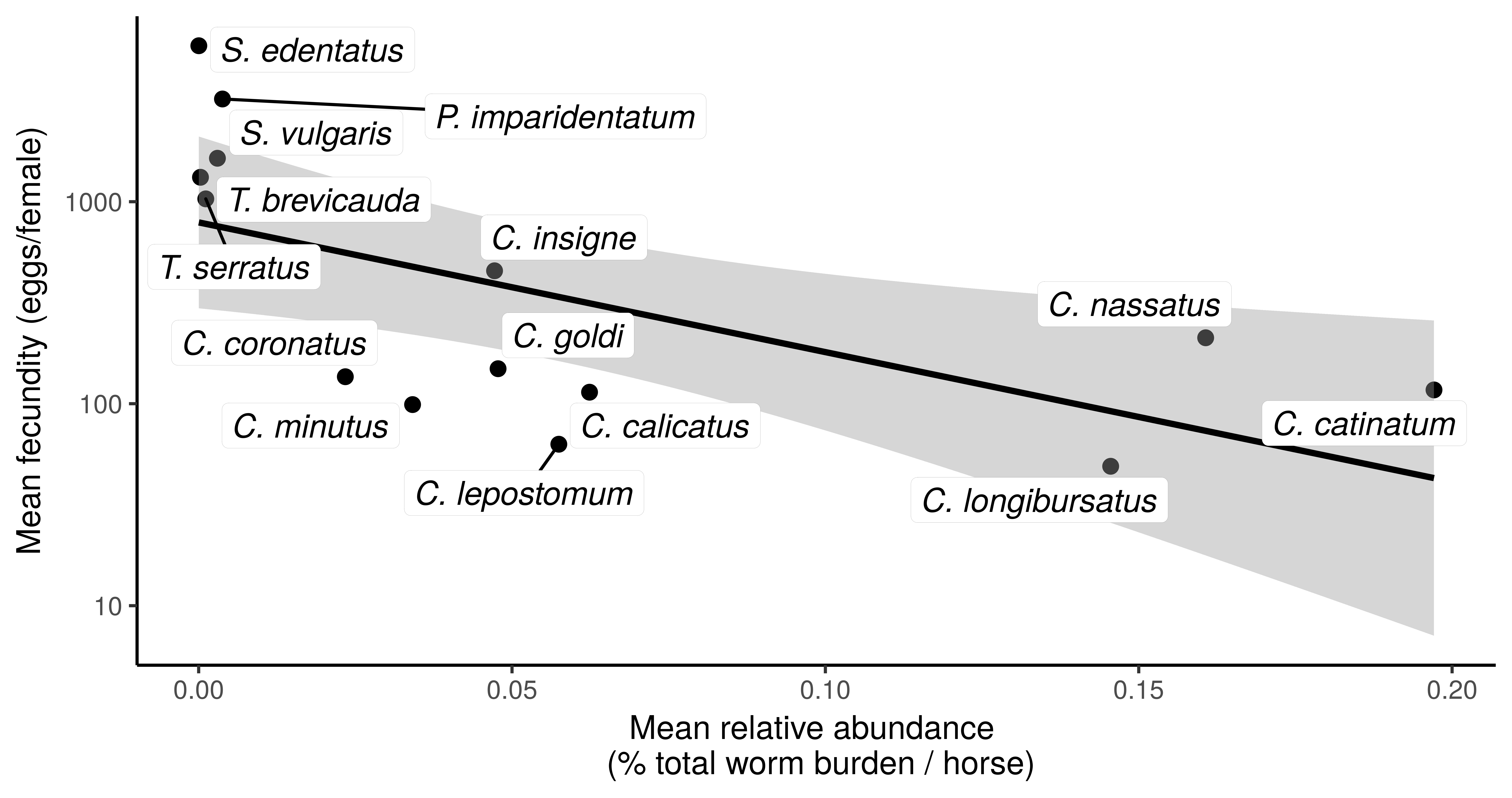

Supplement: Supplementary file 4 — Figure S1. Relationship between observed strongyle abundance and published fecundity estimates. Previously published estimates of strongyle species fecundity (measured as the number of eggs found in female strongyles in utero) are plotted against species relative abundance (measured as the fraction of the total strongyle community within each horse) across monitored horses. Each dot stands for a given strongyle species. The regression line between the two variables is shown as well the 95% confidence interval (grey area). (TIF 737 kb) [file 13071_2018_2858_MOESM4_ESM.tif]
